# The role of centrosomal Nlp in the control of mitotic progression and tumourigenesis

**DOI:** 10.1038/bjc.2011.130

**Published:** 2011-04-19

**Authors:** J Li, Q Zhan

**Affiliations:** 1 State Key Laboratory of Molecular Oncology, Cancer Institute, Chinese Academy of Medical Sciences and Peking Union Medical College, Beijing, People’s Republic of China

**Keywords:** centrosome, cell cycle, ninein-like protein, Nlp, mitosis, tumourigenesis

## Abstract

The human centrosomal ninein-like protein (Nlp) is a new member of the *γ*-tubulin complexes binding proteins (GTBPs) that is essential for proper execution of various mitotic events. The primary function of Nlp is to promote microtubule nucleation that contributes to centrosome maturation, spindle formation and chromosome segregation. Its subcellular localisation and protein stability are regulated by several crucial mitotic kinases, such as Plk1, Nek2, Cdc2 and Aurora B. Several lines of evidence have linked Nlp to human cancer. Deregulation of Nlp in cell models results in aberrant spindle, chromosomal missegregation and multinulei, and induces chromosomal instability and renders cells tumourigenic. Overexpression of Nlp induces anchorage-independent growth and immortalised primary cell transformation. In addition, we first demonstrate that the expression of Nlp is elevated primarily due to *NLP* gene amplification in human breast cancer and lung carcinoma. Consistently, transgenic mice overexpressing Nlp display spontaneous tumours in breast, ovary and testicle, and show rapid onset of radiation-induced lymphoma, indicating that Nlp is involved in tumourigenesis. This review summarises our current knowledge of physiological roles of Nlp, with an emphasis on its potentials in tumourigenesis.

The centrosome serves as the major microtubule organising centre (MTOC) in metazoan cells that regulates cell polarity, adhesion and motility in interphase, and facilitates microtubules (MTs) organisation during mitosis. At the onset of mitotic phase, each centrosome, consisting of a pair of centrioles embedded in an amorphous pericentriolar material (PCM), undergoes striking structural and functional reorganisation, known as centrosome maturation (Bornens, 2002; Bettencourt-Dias and Glover, 2007). This event is principally characterised by recruitment of *γ*-tubulin containing complexes to the centrosome and concomitant increase in MT nucleation capacity, which requires substantial exchange of critical PCM components (Khodjakov and Rieder, 1999). Several protein kinases and phosphatases including Polo-like kinase 1 (Plk1), Aurora A and Nek2 kinases, as well as protein phosphatase 4 have been implicated in the regulation of centrosome maturation (Lane and Nigg, 1996; Fry *et al*, 1998; Sumiyoshi *et al*, 2002), but the substrates of these enzymes for centrosome maturation are largely unidentified.

The recently identified human centrosomal ninein-like protein (Nlp) has been characterised as a novel centrosomal protein that is implicated in centrosome maturation by recruiting *γ*-TuRCs during interphase. Further analysis indicated it as a substrate of several essential mitotic kinases, including Plk1, Nek2, Cdc2 and Aurora B (Bornens, 2002; Casenghi *et al*, 2003, 2005; Rapley *et al*, 2005; Yan *et al*, 2010; Zhao *et al*, 2010). It has been revealed by both our study and Nigg EA *et al*'s work that Nlp needs to be displaced from maturing centrosomes at G2/M transition and that the phosphorylation of Nlp at G2/M transition can disassociates it with the centrosome, which is a critical step for centrosome maturation and mitotic spindle formation. The persistent expression of Nlp can form large aggregation at the centrosome which in turn induces mitotic spindle aberrations, while depletion of Nlp hinders organisation of mitotic spindle at prophase within original mononucleated cell, which results in lagging chromosomes, and micronucleated or multinucleated phenotypes (Casenghi *et al*, 2003; Jin *et al*, 2009; Zhao *et al*, 2010). We showed that centrosomal Nlp is tethered to the interphase centrosome by tumour suppressor BRCA1, which is required to ensure normal MT nucleation at interphase (Jin *et al*, 2009). Moreover, we have found that Nlp phosphorylation by Aurora B is essential to the completion of cytokinesis. Disturbance of these functional cellular processes can induce mitotic spindle aberrations, spindle checkpoint defects, chromosomal missegregation and cytokinesis failure, which may not be sufficient to initiate tumourigenesis, but can induce perpetual state of chromosomal instability (CIN) that facilitates tumourigenesis (Thompson and Compton, 2008; Thompson *et al*, 2010). In spite of CIN caused by abnormal expression of Nlp in cell models, the deregulated expression and genetic alterations of Nlp in human tumours remain unclear. Our previous study also showed that Nlp is frequently overexpressed in several types of human cancers, including breast, lung, ovarian, head and neck cancers. The deregulated expression of Nlp in human tumours is associated with its gene amplification. We first demonstrated that Nlp is an oncogenic protein, the overexpression of which allowed for anchorage-independent growth and induces cell malignant transformation (Qu *et al*, 2008; Yu *et al*, 2009; Shao *et al*, 2010). In Nlp transgenic mice, we revealed that elevated expression of Nlp results in spontaneous tumours and centrosome amplification in mouse embryonic fibroblast (MEF) cell. The Nlp transgenic mice were also prone to DNA-damage and carcinogen-induced tumourigenesis (Shao *et al*, 2010). Although more unequivocal large-scale and long-term consequences of Nlp-null mice models are awaited, sufficient information is now available to review the collective data of oncogenic propensities of Nlp.

## Identification of two novel members of Nlps

*Xenopus* centrosomal Nlp (X-Nlp) was initially identified as a substrate protein of Plx1 by the yeast two-hybrid approach (Casenghi *et al*, 2003). The first human homologue of X-Nlp is identified as a 156-kDa protein. As its N-terminus shares 37% sequence identity to the corresponding domain of MT minus-end-anchoring protein ninein, it is named as Nlp.

All Nlp members share similar structures of C-terminus with variable numbers or sizes of coiled-coil domains that typify most centrosome proteins. It has been indicated that the coiled-coil domains are responsible for interaction with several essential centrosome proteins, such as BRCA1, Aurora B and CDC2 (Jin *et al*, 2009; Yan *et al*, 2010; Zhao *et al*, 2010). The major functions of Nlp are regulated by protein–protein interaction and post-translational regulation. For instance, phosphorylated Nlp at G2/M transition should be displaced from the centrosome to ensure MT nucleation and spindle formation (Rapley *et al*, 2005). The conserved N-terminus of Nlp is predicted to form putative EF-hand motifs that are involved in the interaction with *γ*-tubulin and are essential for centrosome maturation and MT outgrowth (Casenghi *et al*, 2003). Both X-Nlp and human Nlp are predominantly present in the mother centriole and basal body of primary cilia in cultured cells, suggesting that Nlp might contribute not only to MT nucleation but also to MT anchorage (Rapley *et al*, 2005).

It is interesting to mention that the human Nlp comprises at least two splicing variants, Nlp isoform A and Nlp isoform B, encoded by 1382aa and 1033aa, respectively. The Nlp isoform B was recently identified as a novel component of Usher interactome. According to slight structural differences with Nlp isoform A, Nlp isoform B might function as a molecular hinge between intracellular trafficking and intraflagellar transport machinery, suggestive of a novel role for *NLP* gene in physiological photo-reception process and retinal ciliopathies (van Wijk *et al*, 2009).

## The roles of Nlp in mitotic events

**Figure 1:**
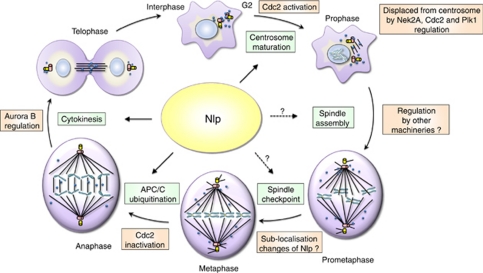
Cell-cycle functions and localisations of Nlp. Nlp associates with multiple structures at multiple stages of cell division. DNA/chromosomes are marked in blue, microtubules in black line and centrosomes in a pair of cylinders (red one represents mother centriole, and bright yellow one, daughter centriole). The blue circles are used to indicate the association of Nlp with different structures.

In mammalian cells, Nlp (also named as Nlp isoform A by van Wijk *et al*) is expressed in a cell-cycle-dependent manner with a peak at G2/M transition. Its subcellular localisation undergoes dynamic changes during mitosis. Further analysis suggests that Nlp, with a relatively short half-life, is able to be ubiquitinated and degraded via APC/C pathway (Wang and Zhan, 2007). The fluctuating expression and localisation patterns suggest that Nlp is temporally and spatially involves in various mitotic events, such as centrosome maturation, spindle formation and cytokinesis (Casenghi *et al*, 2003, 2005; Rapley *et al*, 2005; Yan *et al*, 2010) (Figure 1).

### Nlp, a new *γ*-tubulin complex binding protein, physically associated with BRCA1, functioning in MT nucleation

In the majority of animal cells and yeast, most MTs are nucleated from MTOC, notably centrosomes and spindle pole bodies, respectively. This process primarily depends on the recruitment of *γ*-tubulin-containing multiprotein complexes, which are only activated after their recruitment to the MTOC (Schiebel, 2000; Bettencourt-Dias and Glover, 2007). But the mechanism how these complexes are recruited to the centrosome is still uncovered. In most cases, *γ*-tubulin complex binding proteins (GTBPs) are thought to act like receptors for *γ*-tubulin complexes and aid MT nucleation (do Carmo Avides and Glover, 1999). So far only a few GTBPs have been identified, either in lower organisms or in vertebrates, notably Spc72p and Spc110p in *Saccharomyces cerevisiae*, Abnormal spindle protein in Drosophila and kendrin/pericentrin/CG-NAP in mammalian cells (Francis and Davis, 2000; Schiebel, 2000). Casenghi *et al* (2003) initially reported that Nlp, a novel candidate GTBP, can bind to *γ*-tubulin and hGCP4. By recruiting the entire *γ*-TuRCs, Nlp assemblies can stimulate MT asters in both *xenopus* egg extracts and mammalian cells. In 2005, Casenghi *et al* found that dynein–dynactin complex was required for targeting Nlp to the centrosome. This process is negatively regulated by Plk1, which promotes the release of centrosomal Nlp by phosphorylation and prevents re-supply of Nlp to the centrosome by dynein–dynactin motor complex. Additionally, they suggested that MTs are required for this recruitment of newly synthesised Nlp, but not for the maintenance of Nlp at the centrosome. The observations above raise the question how is Nlp maintained and regulated at the centrosome during interphase (Casenghi *et al*, 2005).

It is worth noting that we have recently characterised Nlp as a BRCA1-associated protein which co-localises with BRCA1 at the centrosome during interphase. Functional BRCA1 regulates Nlp centrosomal localisation and protein stability through inhibition of Plk1. EGFP-Nlp in the cells depleted of BRCA1 show large aggregation in cytoplasm and increasing susceptibility of degradation as BRCA1 can elevate expression of Plk1. Thus, besides interaction with *γ*-tubulin and hGCP4, the association of Nlp with BRCA1 at interphasic centrosome is essential for the completion of MT nucleation activity and spindle organisation (Jin *et al*, 2009).

It was reported that abolishing endogenous Nlp expression inhibits MT nucleation and spindle formation *in vitro* (Casenghi *et al*, 2003). Moreover, the depletion of Nlp hinders organisation of mitotic spindle at prophase within original mononucleated cells, which in turn results in chromosomal missegregation and multinucleated phenotypes (Jin *et al*, 2009). On the other hand, overexpression of wide-type Nlp causes aberrant spindles in mitotic cells, including monopolar, tripolar or tetrapolar spindles (Casenghi *et al*, 2003). These observations suggest that normal abundance of Nlp is required for mitotic progression, and both upregulation and downregulation of Nlp during mitosis perturb spindle formation.

### Coordinated regulation of Nlp by mitotic kinases Cdc2, Nek2A and Plk1

Nlp serves as one of the substrates of Plk1 (Casenghi *et al*, 2003). Overexpression of a constitutively active mutant of Plk1 (Plk1**^T210^**D) can induce premature dissociation of Nlp and *γ*-TuRCs during centrosome maturation. Complete disassembly of Nlp from the centrosome requires phosphorylation at multiple sites, EDS^87^S^88^SLE, ST^161^KEA, and EKS^686^QEV, which conform to an E/DxS/T consensus motif of Plk1 substrates. Cells co-expressing NlpΔ8 mutant (with all eight plk1 phospho-sites mutated to alanine) and Plk1**^T210^**D are tremendously resistant to fragmentation of centrosomal Nlp into cytoplasm, which in turn contributes to false spindle formation. Thus, it is tempting to raise a possibility how Nlp functions in mitosis. First, Nlp may recruit *γ*-TuRCs to the centrosome at interphase. Upon entry into mitosis, centrosomal Nlp is phosphorylated by Plk1 and displaced from centrosome, which is a prerequisite for correct spindle formation.

**Figure 2:**
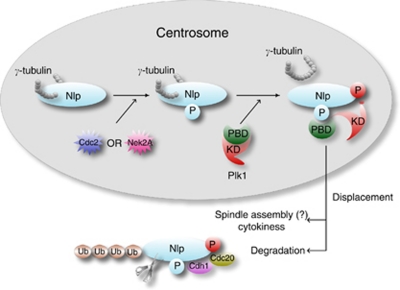
A schematic diagram for the coordinated regulation of Nlp by mitotic kinases Cdc2, Nek2A and Plk1. At interphase, Nlp is involved in docking *γ*-TuRC at the centrosome. While Cdc2 activation at G2 to M transition initiates the activation of Nek2A, Nlp can be phosphorylated by these two priming kinases, which creates docking sites for PBD of Plk1. Upon binding to primary phosphorylated sites through PBD, Plk1 becomes partially active via dissociation of its kinase domain (KD) from PBD. Subsequently, Nlp is subjected to further phosphorylation by Plk1 and is displaced from the centrosome, which facilitates centrosome maturation and spindle assembly.

Intriguingly, according to observations from us and other groups, centrosomal Nlp seems to be modulated in a concerted action by both Cdc2 and Nek2A priming for Plk1 (Rapley *et al*, 2005; Zhao *et al*, 2010). A potential mode how Nlp is sequentially phosphorylated is raised although it needs further confirmation (Rapley *et al*, 2005; Zhao *et al*, 2010) (Figure 2). At the onset of mitosis, Nlp is phosphorylated at Ser185 by Cdc2, creating a docking site for PBD of Plk1. Ninein-like protein is further phosphorylated by Plk1 at four putative sites with PBD recognition consensus, EDS**^87^**S**^88^**SLE, ST**^161^**KEA and EKS**^686^**QEV, leading to Nlp oligomerisation into cytoplasm (Casenghi *et al*, 2003; Park *et al*, 2010). Mutants with Cdc2 phospho-sites show compromised, but not totally abolished fragmentation by Plk1. Intriguingly, some evidence suggests that centrosomal Nlp seems to be primarily phosphorylated not only by Cdc2 but also by Nek2A kinase. One possibility is that, with absence of Cdc2 priming phospho-sites, Nlp is primed by Nek2A at unidentified but distinct phospho-sites, promoting its phosphorylation by Plk1 to achieve centrosome maturation (Rapley *et al*, 2005). This suggests that Nek2A might be an alternative priming kinase of Plk1 for Nlp. Kinase-dead mutant Nek2A strongly interferes with the ability of Plk1 to diminish centrosomal Nlp staining but not vice versa, thus putting Nek2A upstream of Plk1 in Nlp regulation network. It is also reported that interphasic Nek2A is inhibited by PP1. Upon entry into mitosis, PP1 itself is inhibited as a result of binding to the Inhibitor-2 protein (Inh-2), an interaction that may be stimulated by activated Cdc2 (O’Regan *et al*, 2007). Thus, we postulate that Cdc2 activation for G2 to M transition can initiate sequential activation of Nek2A. Coordination of these two kinases might be a new mechanism for Plk1 priming, at least for Nlp.

Collectively, the involvement of Nlp in centrosome maturation includes two steps. Ninein-like protein functions as a docking protein for *γ*-TuRCs, on which MT nucleation primarily depends. Subsequently, Nlp needs to be displaced from the centrosome by coordinated phosphorylation of Cdc2, Nek2A and Plk1 in order to ensure centrosome maturation and mitotic spindle formation (Casenghi *et al*, 2003; Rapley *et al*, 2005; Zhao *et al*, 2010). Concerning the regulation modes of Nlp initiated by either priming kinases, Cdc2 or Nek2A for Plk1, we speculate that these two seemly redundant phosphorylation modes may not mutually exclusive and may work in a concerted manner in particular scenarios.

### Recurrence of Nlp in cytokinesis

As the centrosome is involved in positioning of central spindlin and midbody to ensure equal abscission and completion of cytokinesis (Schuyler and Pellman, 2001), a question is raised whether centrosomal Nlp is somehow implicated in this process. As far as we are concerned, depletion of Nlp triggers asymmetric division and leads to multinucleated phenotype, similar to its overexpression as previously described (Wang and Zhan, 2007). Even if Nlp malfunction in MT nucleation and spindle formation undoubtedly fosters multinucleated progenies, we still need to explore its potential direct contributions to cytokinesis. Recently, we found that endogenous Nlp recurred at the midbody region during cytokinesis, though not so prominent as that at interphasic centrosome (Yan *et al*, 2010) (Figure 1). Moreover, Nlp could also bind to the CPC complex through interacting with Aurora B kinase (the core component of the CPC complex). Disrupting their interaction resulted in cytokinesis failure. Bioinformatics analysis of Nlp protein sequence showed that it has consensus phosphorylation motif ((R/K)_1−3_X(S/T)) for Aurora B (Meraldi *et al*, 2004) and mapped the specific phospho-sites to Ser185, Ser448 and Ser585. A series of evidence demonstrated that Nlp is a novel substrate of Aurora B and the phosphorylation at Ser448 and Ser585 of Nlp is likely required for its association with Aurora B in midbody. Mutations of Nlp-S448A and Nlp-S585A result in fragmentation of Nlp as well as impaired cytokinesis. Meanwhile, phosphorylation at Ser185 of Nlp is of considerable importance, not only for stabilising the recruitment of Nlp signal by Aurora B, but also for ensuring no premature destruction of Nlp before completion of cytokinesis (Yan *et al*, 2010).

Significantly, phosphorylation of Nlp at all three sites by Aurora B does not affect its interaction with *γ*-tubulin in midbody. This phenomenon is coincidently consistent with the classical assertion that the final events of cell cleavage are correlated with the movement of the mother centriole to the intercellular bridge, midbody, at the end of telophase. This movement coincides with bridge narrowing and MT depolymerisation, in which plenty of MT-associated proteins at the plus end and motor proteins are involved (Piel *et al*, 2001). Accordingly, we presume a necessity for the recruitment of Nlp by Aurora B to midbody to fulfil the final task. As a platform, Nlp transiently stimulates MT nucleation with *γ*-tubulin and coordinately organises other undefined elements to maintain the stability of midbody apparatus. Any aberrations of Nlp during cytokinesis would induce CIN and facilitate tumourigenesis.

## Implications of centrosomal NLP in tumourigenesis

The human centrosomal *NLP* gene (KIAA0980) maps to chromosome 20p11, a region frequently amplified in extensive epithelial malignancies (Larramendy *et al*, 2000). Based on the prevailing theory, centrosome aberrations are presumed to facilitate tumourigenesis by fostering aberrant spindle, which consequently results in enhanced CIN and cytokinesis failure. Since Nlp has a fundamental role in MT nucleation, spindle formation and cytokinesis, and is subjected to a complex post-modification by mitotic kinases, its proper abundance with correct phosphorylation state at each mitotic phase is a prerequisite for precise execution of the mitotic events and cell division (Casenghi *et al*, 2003, 2005; Jin *et al*, 2009; Yan *et al*, 2010; Zhao *et al*, 2010). These results leave the possibility open that Nlp may be deregulated in human tumours. We found that the expression of Nlp is elevated in breast and lung carcinomas, which is mostly correlated with its gene amplification. We also found the oncogenic potentials of Nlp, which allow anchorage-independent growth and cell transformation. Consistently, the transgenic mice overexpressing Nlp exhibit centrosome amplification, spontaneous tumours in late stage of life and susceptibility to DNA-damage and carcinogen-induced tumourigenesis (Shao *et al*, 2010 and unpublished observations). Given that the underlying machinery remains obscure, it is still beneficial to delineate the hot spots of oncogenic potential of *NLP* gene based on our preliminary perspectives.

### Overexpression and gene amplification of Nlp in human cancers

Our recent studies have shed dim light on the correlation of overexpression of Nlp with clinical outcomes. It has been showed that Nlp is frequently overexpressed in extensive epithelial malignant tumours, for instance, 80% breast cancer tissues, 65.8% of head and neck squamous cell carcinoma (HNSCC) and 78% of lung cancer (Qu *et al*, 2008; Yu *et al*, 2009). Importantly, the level of Nlp in ovarian cancer concurs with tumour grade, albeit not with FIGO stage or histological types. It is concomitantly elevated from benign, borderline to malignant ovarian tissues (Qu *et al*, 2008). Consistently, accumulated evidence suggested that Nlp overexpression in HNSCC is initiated as early as in premalignant lesions, particularly in preinvasive dysplasia yet to acquire integral malignancy. These findings may indicate that Nlp overexpression is an early event in tumourigenesis (Yu *et al*, 2009).

To further investigate the underlying mechanism that Nlp's upregulation promotes tumourigenesis, 30 paired clinical lung cancer tissues were analysed, and samples overexpressing mRNA and protein of Nlp occupied 60% (Shao *et al*, 2010). Remarkably, it is found that about 40% *NLP* (KIAA0980) genes amplified in 30 primary lung carcinomas contrast to their normal adjacent counterparts by either genomic southern blot assay or fluorescence *in situ* hybridisation. Likewise, *NLP* (KIAA0980) gene amplification was also observed in breast and lung cancer cells. These findings strongly suggest that there are genetic alterations of *NLP* during tumourigenesis.

Nevertheless, the discrepancy between gene amplification and overexpression has long been observed. One representative case is that amplification of *STK15/aurora-a* is seen in only 3% of hepatocellular carcinomas (HCCs), while >60% of HCCs overexpress Aurora A mRNA and protein (Jeng *et al*, 2004). One possible reason is that except for gene amplification, overexpression of Aurora A is also attributable to extenuated protein degradation and transcriptional activation. Similarly, it has been reported that Nlp mutants harbouring KEN-box and D-box mutations or respective phosphorylation-sites mutations of Plk1, Cdc2 or Aurora B kinases show resistance to proteolysis (Casenghi *et al*, 2003; Wang and Zhan, 2007; Yan *et al*, 2010; Zhao *et al*, 2010). This might may hold true in human malignancies. Another possibility is that Nlp may be upregulated at the transcription level by oncogenic transcription factors. The recent comparative transcriptome profiling of invasive breast carcinoma based on ERα status has identified *NLP* (KIAA0980) as a novel oestrogen responsive genes (Abba *et al*, 2005). It contains a putative high-affinity oestrogen responsive elements mapping in proximity to transcription starting sites, suggesting *NLP* (KIAA0980) transcription may be directly under hormonal control. Undoubtedly, cross-referencing these phenotypes with Nlp augmentation in human malignancies might be more informative. Hence, *NLP* (KIAA0980) gene amplification is not enough to account for all cases of overexpression of Nlp protein in human tumours. The defect of Nlp degradation process and *NLP* transcriptional regulation might also contribute to deregulated expression of Nlp in human cancers.

### Oncogenic potential of Nlp in immortalised rodent fibroblasts and transgenic mice

Transformation of immortalised primary cells has been considered as a standard criterion to determine the oncogenic propensity of an unclassified gene (Perucho *et al*, 1981). Several notable oncogenes were initially identified by *in vitro* transformation assay (Chang *et al*, 1982). Intriguingly, immortalised rodent fibroblasts NIH3T3 overexpressing Nlp displayed anchorage-independent growth and tumour formation in nude mice (Shao *et al*, 2010). These findings suggest that Nlp may be a potential oncogenic protein.

The oncogenic potentials of Nlp were further exhibited in transgenic mice (Shao *et al*, 2010). Although the mice with elevated expression of Nlp were viable and fertile, and were apparently normally developed, the animals were able to develop spontaneous tumourigenesis, including both primary and metastatic tumours. Interestingly, four male mice developed primary intratubular germ cell carcinoma, while another four female animals were prone to breast ductal carcinoma. Besides, one female was found to have ovarian adenocarcinoma metastasis in the right hind leg and lung tissues. In addition, Nlp transgenic mice revealed more rapid onset of radiation-induced lymphomas, most of which display invasive behaviours to neighbouring organs like heart and lung. Conversely, the lymphomas generated in approximate half of control animals are largely confined to the thymus and rarely invaded the neighbour organs. Particularly, both MEFs and glomerular cells derived from Nlp transgenic mice exhibited frequent centrosome amplification, suggesting that Nlp overexpression is tumour prone, which mimics BRCA1 loss *in vivo* (Xu *et al*, 1999).

It is well established that supernumerary centrosomes can arise from diverse mechanisms (Doxsey, 2001). One is centriole reduplication in a single S phase which is previously regarded as upon loss of G1/S checkpoint, DNA-repair pathway such as loss of p53 or BRCA1 (Fukasawa *et al*, 1996; Deng, 2002). Moreover, *de novo* centrosome formation also contributes to aberrations of centrosome numbers (Khodjakov *et al*, 2002). More recently, our knowledge has been further updated on how procentrioles is induced to maturation and dissociated with mother centrioles in the presence of activated Plk1**^T210^**D at the centrosome (Loncarek *et al*, 2010). Generally, we cannot rule out the possibility that deregulated Nlp may have a role in centriole reduplication as it interacts with BRCA1 during the whole interphase (Jin *et al*, 2009). Another two mechanisms for centrosome amplification are multipolar spindle formation and cytokinesis failure, which are apparently manifested in elevated expression of Nlp as discussed above (Casenghi *et al*, 2003; Yan *et al*, 2010). Remarkably, both of these phenotypes were exacerbated in the Nlp transgenic mice (Shao *et al*, 2010). Therefore, even though available evidence is not sufficient to favour one machinery over another for centrosome amplification in Nlp transgenic mice, by no means, the importance of deregulated centrosomal Nlp to fuel tumour progression is diminished, as supernumerary centrosomes always set a stage for CIN and tumourigenesis, regardless of their origins.

## Conclusions and future perspectives

Better understanding of how centrosomes contribute to cellular functions requires the isolation and characterisation of unknown centrosome-associated molecules. Here, we have characterised a novel human centrosomal component Nlp and preliminarily defined its oncogenic potential in tumourigenesis. So far several lines of evidence have linked Nlp to human cancers (Shao *et al*, 2010). The *NLP* (KIAA0980) gene is amplified in a broad range of primary tumours and tumour-derived cell lines. Moreover, overexpressing Nlp can transform primary cells *in vitro* and initiate tumour formation in nude mice. Transgenic mice overexpressing Nlp also display spontaneous tumourigenesis in the breast, ovary and testicle, and rapid onset of radiation-induced lymphoma, indicating its involvement in tumourigenesis.

Nonetheless, the picture remains to be completed. Multiple provocative issues need to be addressed in the future. First of all, it will be vital to further explore the intricate regulatory network of Nlp by other kinases and phosphatases, which may determine the additional roles of Nlp during mitosis or biological events. Second, considering the essential role of Nlp isoform B in ciliopathies (van Wijk *et al*, 2009), equally possible is that Nlp has unidentified conserved functions in ciliary biology and human pathologies, such as in certain recessive diseases. Prominently, several recent studies strongly support the unexpected links of ciliopathies and carcinogenesis (Han *et al*, 2009; Nigg and Raff, 2009; Wong *et al*, 2009); leaving open the possibility that Nlp isoform B, similar to its sibling Nlp isoform A, may be also involved in some unidentified oncogenic events. Moreover, much remains to be learned about whether the transcriptional activity of *NLP* (KIAA0980) gene is under hormone control hinted by transcriptome studies of breast carcinoma (Abba *et al*, 2005). This undoubtedly represents an intriguing task for better understanding of breast carcinogenesis. Collectively, it is strongly suggested that overexpression of centrosomal Nlp might be a critical early event in tumour development, or rate-limiting step for acquisition of invasive properties by neoplastic cells. So another upcoming task should be aimed at interpreting whether the phenotype, that is, the displacement of Golgi apparatus from the centrosome by upregulation of Nlp or ninein can interfere with cell migration and cell polarisation that require a high coordination of the centrosome and the Golgi apparatus. (Casenghi *et al*, 2005). Therefore, dissecting the versatile roles of centrosomal Nlp members in human disorders is still a worthwhile challenge ahead.
